# Erratum to: Ventilator-associated pneumonia in the ICU

**DOI:** 10.1186/s13054-016-1206-8

**Published:** 2016-01-28

**Authors:** Atul Ashok Kalanuria, Wendy Ziai, Marek Mirski

**Affiliations:** 1 Department of Neurology, University of Maryland School of Medicine, Baltimore, MD 21201 USA; 2 Department of Anesthesiology/Critical Care Medicine, Johns Hopkins University School of Medicine, Baltimore, MD 21287 USA

After publication of this article [1] it has been found that the name of the second author has been misspelt. The correct name is Wendy Ziai which has been herewith corrected in this erratum.
